# Multilocus Genotyping of ‘*Candidatus* Phytoplasma Solani’ Associated with Grapevine Bois Noir in Iran

**DOI:** 10.3390/biology11060835

**Published:** 2022-05-29

**Authors:** Elham Jamshidi, Sergio Murolo, Sareh Baghaee Ravari, Mohammad Salehi, Gianfranco Romanazzi

**Affiliations:** 1 Department of Agricultural, Food and Environmental Sciences, Marche Polytechnic University, I-60131 Ancona, Italy; e.jamshidi@staff.univpm.it (E.J.); s.murolo@univpm.it (S.M.); 2 Plant Protection Department, Ferdowsi University of Mashhad, Mashhad 1696700, Iran; s.baghaee@um.ac.ir; 3 Plant Protection Research Department, Fars Agricultural and Natural Resources Research and Education Centre, AREEO, Zarghan 73415-111, Iran; salehi_abarkoohi@yahoo.com

**Keywords:** genetic variability, molecular epidemiology, phytoplasma, *Vitis vinifera*

## Abstract

**Simple Summary:**

Understanding the epidemiological cycle of Bois noir (BN) associated with ‘*Candidatus* Phytoplasma solani’ is vital to managing the disease effectively. The genotyping of ‘*Ca.* P. solani’ strains, according to *tuf*, *vmp*1, and *stamp* genes, contributes to a better knowledge of the geographical distribution of BN. In this study, we provide information on the molecular variants of ‘*Ca*. P. solani’ in Iranian vineyards. We observed six known *vmp*1 variants and discovered two new ones, V24 and V27. This information is useful for future investigations to more accurately understand the epidemiological cycle(s) of BN in Iranian vineyards, contributing to the management of the disease.

**Abstract:**

Grapevine Bois noir (BN) is associated with ‘*Candidatus* Phytoplasma solani’. It has been recorded in vineyards throughout Europe as well as in different countries in Asia, where it now constitutes a threat to Iranian viticulture. BN is strictly dependent on ‘*Ca*. P. solani’ strains, wild host plants, and insect vectors. The molecular typing of ‘*Ca*. P. solani’, based on the nonribosomal gene *tuf* and the two hypervariable markers *vmp*1 and *stamp*, is valuable for the reconstruction and clarification of the pathways of BN spread. In this study, an RFLP analysis was performed on the *vmp*1 gene, and a single-nucleotide polymorphism analysis confirmed new *vmp* types in ‘*Ca*. P. solani’. A *stamp* gene phylogenetic analysis allowed us to distinguish between the new genotype infections in the grapevines and the ‘weeds’ *Convolvulus arvensis* and *Erigeron bonariensis* in Iranian vineyards, highlighting the close genetic relatedness of the strains of ‘*Ca*. P. solani’ found in Iran and Azerbaijan. The most common genotype in the grapevines was *tuf* b/V24/*stamp* III, which was associated with *C*. *arvensis*. This information contributes toward the identification of further routes of introduction of ‘*Ca*. P. solani’ in Iran to sustain the control measures for the management of BN.

## 1. Introduction

Grapevine Bois noir (BN) is a ‘*Candidatus* Phytoplasma solani’ (‘*Ca*. P. solani’)-associated disease that causes significant crop losses [1,2,3,4]. BN has been recorded in vineyards throughout Europe as well as in different countries in Asia (e.g., Israel, Jordan, Lebanon, Syria, Iran, Azerbaijan, Turkey, Georgia, Russia, and China) [5,6,7,8,9,10,11,12,13,14]. The epidemiology of ‘*Ca*. P. solani’ is highly correlated with the host plant reservoirs, from which the insect vectors (mainly belonging to the *Cixiidae* family such as *Hyalesthes obsoletus* [15] and *Reptalus panzeri* [16]) acquire a phytoplasma inoculum from feeding.

Over the past 20 years or so, several studies have been published regarding potential vectors [17,18,19,20,21]. Recently, Quaglino et al. [22] demonstrated that nine new insect vectors could transmit ‘*Ca.* P. solani’, which has raised several questions regarding the biological cycle of ‘*Ca.* P. solani’.

Multilocus sequence typing (MLST) analyses of nonribosomal genes have been widely applied to molecular epidemiology [23,24,25,26]. The molecular typing of ‘*Ca*. P. solani’ isolates associated with BN is important to clarify the pathways of BN spread [27,28,29,30]. The study of the molecular epidemiology of ‘*Ca*. P. solani’ isolates considers the genetic diversity of the different molecular typing markers. Among these, the most widely used are the *tuf* gene, which encodes the translation elongation factor TU [27]; the *stamp* gene, which encodes the antigenic membrane protein AMP [30]; and the *vmp*1 gene, which encodes a variable membrane protein [31,32].

According to the *tuf* gene characterization, ‘*Ca*. P. solani’ isolates are grouped into two main genotypes, *tuf*-a and *tuf*-b, which were shown to be related to the different natural epidemiological cycles of ‘*Ca*. P. solani’ in western Germany [24]. Isolates of the *tuf*-a type were spread from the stinging nettle (*Urtica dioica*), but isolates of the *tuf*-b type were disseminated from bindweed (*Convolvulus arvensis*). A third minor genotype, *tuf*-c, was shown to propagate from the bindweed *Calystegia sepium* [26,27]. Recently, different *tuf*-b sub-genotypes (molecular variants) have been proposed to be involved in an epidemiological cycle of BN [29,33,34]. Sequence analyses of *vmp*1 and *stamp*, which are variable genes, have provided evidence of a high variability among the BN strains in terms of the *tuf* types [35,36]. Based on *Rsa*I RFLP and sequencing analyses of partial *vmp*1 genes, 25 *vmp* molecular types of ‘*Ca*. P. solani’ were described in vineyard ecosystems around the world [35]. Based on the phylogenetic analyses of the concatenated nucleotide sequences of the genes *vmp*1 and *stamp* for 76 ‘*Ca*. P. solani’ strains, 49 *vmp*1/*stamp* sequence variants were then grouped into 5 *vmp*1/*stamp* clusters [37]. The *vmp*1*/stamp* IV cluster included ‘*Ca*. P. solani’ (*tuf* type a) and was associated with the nettle-related biological cycle, whereas the other four clusters, that included ‘*Ca*. P. solani’ (*tuf* type b), were associated with the bindweed-related biological cycle. Recent studies and continuous surveys from vineyards in Iran have highlighted a significant increase in BN and, consequently, considerable yield losses based on production quantity [38]. To select effective control strategies, comprehensive investigations into BN to clarify the ambiguous aspects of the disease and the epidemiological cycle are critical. Due to the complexity of BN, the numerous weeds associated with vineyards, and the lack of detailed investigations into the weeds in vineyards, we performed the molecular typing of ‘*Ca*. P. solani’ isolates associated with grapevines and the most prevalent weeds in vineyards in this study.

## 2. Materials and Methods

### 2.1. Sample Collection and DNA Extraction

From August to October 2016, 2017, and 2018, 225 BN-symptomatic grapevine leaf samples and 100 symptomatic and asymptomatic weeds (*Convolvulus arvensis* and *Erigeron bonariensis*) were collected (the detailed database is listed in Supplementary Material Table S1) and the molecular detection and further multilocus sequence typing of ‘*Ca*. P. solani’ isolates were undertaken (Table 1). The grapevine and herbaceous samples were collected from the seven main grapevine-growing provinces in Iran—Azarbaijan Gharbi, Azarbaijan Sharghi, Zanjan, Qazvin, Fars, Lorestan, and Khorasan Razavi—from the same areas reported by Jamshidi et al. [39]. Asymptomatic plant materials were also collected as negative controls. The total DNA was extracted from the petioles of the grapevine leaf samples and the roots of the weeds using the CTAB DNA extraction procedure [39].

### 2.2. Molecular Typing and Phylogeny of ‘Candidatus Phytoplasma solani’ Strains

A preliminary analysis was performed on the 225 collected samples of grapevine and the 100 collected samples of weeds using the universal P1/P7 primer pair followed by a nested PCR with rStol/fStol-specific primers (Table S2) [40]. The positive samples were subjected to further molecular typing based on *tuf*, *vmp*1, and *stamp* hypervariable genes. The *tuf* gene was amplified using the STOLTUF-F0/STOLTUF-R0 primer pair for a direct PCR [33] and the TufAYf/r primers for a nested PCR following Langer and Maixner [27]. For *vmp*1, amplification was performed with the primer pair StolH10F2/R2 [16], followed by the primer pair TYPH10F/R [41]. A PCR-RFLP analysis was performed with *Rsa*I. The digested fragments were visualized on 2.5% agarose gels. The *stamp* gene was amplified by a nested PCR with the primer pair StampF/R0 followed by StampF1/R1, as per Fabre et al. [42]. The amplified *tuf*, *vmp*1, and *stamp* genes were sequenced in both directions by a genomics sequencing service (Genewiz UK, Takeley, UK; https://www.genewiz.com/ accessed on 22 May 2022). These Iranian *vmp*1 and *stamp* sequences were compared with the sequences available in the database. For the *vmp*1 sequences, a double-check was performed using a virtual RFLP analysis by applying the pDRAW32 software (http://www.acaclone.com/ accessed on 22 May 2022), which performs a restriction analysis based on the nucleotide sequences. For both *vmp*1 and *stamp*, the nucleotide sequences were used for the phylogenetic analysis. Multiple sequence alignments were identified using CLUSTAL-W software. Using MEGA7 [43], we calculated the phylogenetic relationships. The maximum parsimony trees were obtained with a Subtree–Pruning–Regrafting (SPR) algorithm [44]; the trees were drawn to scale with branch lengths calculated using the average pathway method.

## 3. Results

### Molecular Typing of ‘Candidatus Phytoplasma solani’ Strains Based on the Tuf, vmp1, and stamp Genes

**Figure 1:**
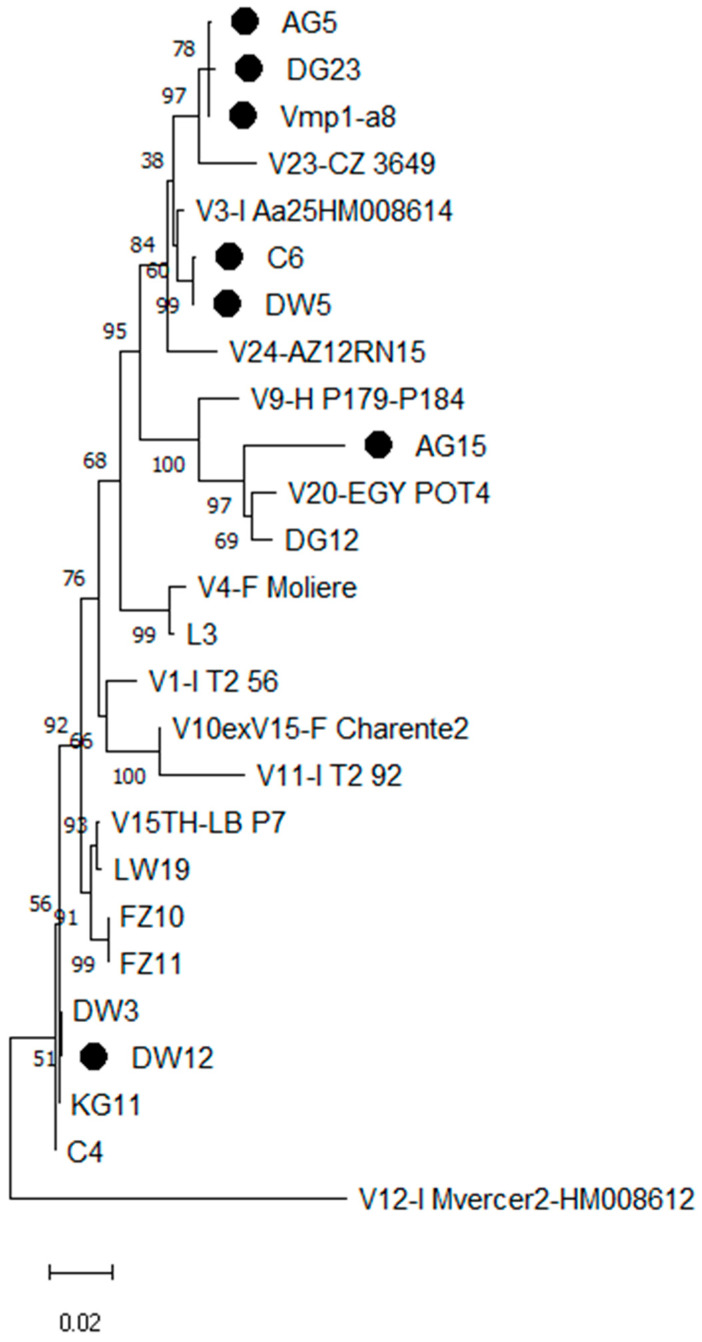
Phylogenetic tree inferred from the ‘*Candidatus* Phytoplasma solani’ strain nucleotide sequences of the *vmp*1 gene. The evolutionary history was inferred using the maximum parsimony method. The MP tree was obtained using the Subtree–Pruning–Regrafting (SPR) algorithm. The percentage of replicate trees in which the associated taxa clustered together in the bootstrap test (500 replicates) are shown next to the branches. The names of phytoplasma strains included in the phylogenetic analysis are given in the tree image. The GenBank accession numbers of the sequences are given; the gene sequences obtained in our investigation are indicated by filled circles.

From the preliminary analysis performed on 16Sr DNA using the fStol/rStol primer pair in the nested PCR, 218 grapevine and weed samples were defined as infected by ‘*Ca.* P. solani’. According to the *tuf* gene analysis, most of the grapevines and weeds were *tuf* type b1. Strains a8 (*tuf* b5) and DG23 (*tuf* b6) [34] were included in the current study for the molecular typing analysis. The positive samples then underwent a PCR with STOLH10F2/R2 followed by TYPH10F/R primers. Polymorphic amplicons that ranged from 1100 bp to 1250 bp were obtained from approximately 142 grapevine and 27 weed samples, which corresponded with 65% of the samples analyzed. A restriction digestion with the *Rsa*I enzyme allowed the identification of eight *vmp*1 profiles of ‘*Ca.* P. solani’ infecting the grapevines, *C. arvensis*, and *E. bonariensis*. Based on the *vmp*1 typing, the already known types V1, V3, V4, V10, V15, and V20 were found in the Iranian vineyards sampled. Two new additional *vmp* restriction patterns, named V24 and V27, were proposed according to the SEE-ERANET database [35]. These profiles were determined from the actual RFLP analysis on the amplified *vmp*1 gene and confirmed by a virtual RFLP analysis on the *vmp*1 nucleotide sequences (Figure S1). A *vmp*1 gene single-nucleotide polymorphism analysis confirmed the new V24 types for strains a8, AG5, and DG23, which only had two repeated domains. The *vmp*1 sequence of strain AG15 had an additional *Rsa*I site at position 621 and three repeated domains and constituted the new V27 RFLP type. The phylogenetic relationship clustered the strains in this study with the ‘*Ca*. P. solani’ reference strains. Strains C4, DW3, DW12, and KG11 were defined as *vmp*1 type V1; C6 and DW5 were defined as *vmp*1 type V3. Sequences with RFLP type V24—namely, the a8, AG5, and DG23 strains—were grouped in the phylogenetic tree. The *vmp*1 sequence of strain AG15 did not match any reference strain (Figure 1).

**Figure 2:**
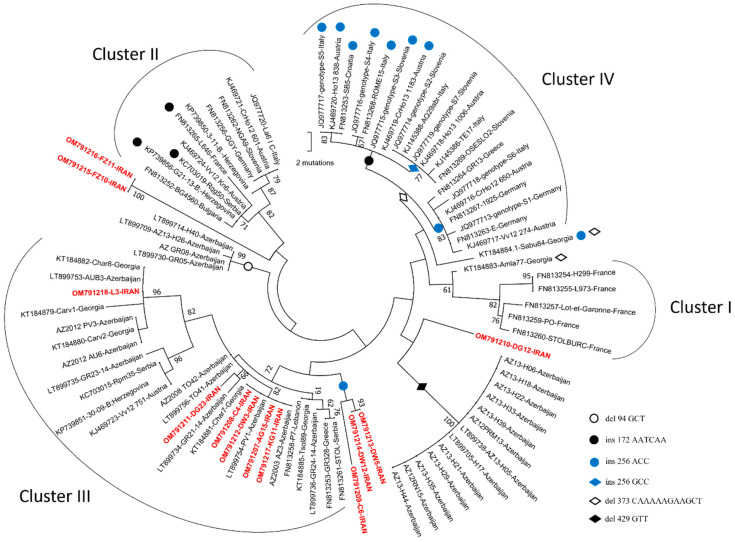
Phylogenetic analysis of the *stamp* sequences using maximum parsimony. Iranian ‘*Candidatus* Phytoplasma solani’ isolates are indicated in red. The most parsimonious tree is shown with the bootstrap values indicated along the branches. Insertions and deletions (see Key, bottom right) for the isolates or phylogenetic branches are indicated at the top of the isolate names or along the branches. The *stamp* genetic clusters are those described in Fabre et al. [42].

A *stamp* gene phylogenetic analysis distinguished six ‘*stamp* clusters’. Four of these have already been reported and belong to clusters I, II, III, and IV [30,42], but two additional molecular clusters were exclusively represented by these Iranian and Azerbaijan ‘*Ca*. P. solani’ strains in different clusters. This was shown by a maximum parsimony phylogenetic analysis. The six *stamp* genotypes detected in the grapevines (KG11, DW3, C4, AG15, L3, and DG23) belonged to *stamp* cluster III. DG12 was unique, with a CAAAAAGAAGCT deletion at position 373 and a CAAAAAGAAGCT deletion at position 361. The DW5, DW12, and C6 genotypes also had a CAAAAAGAAGCT deletion at position 373 and an ACC insertion at position 256 (Figure 2).

## 4. Discussion

Bois noir is one of the most important and dangerous grapevine diseases in the growing areas of Europe and Asia [41]. BN represents a threat to Iranian viticulture, with outbreaks now recorded in different regions in Iran [39]. A recent study defined *vmp* types in grapevines and reservoir plants and identified the *tuf*-b type in Iranian grapevines [34,39]. The epidemiological cycles have not yet been fully elucidated for Iranian vineyard ecosystems.

Considering the complexity of the disease as well as different insect vectors, numerous different weeds as reservoir sources in vineyards, and the vast viticultural areas in the country, we collected and analyzed different grapevine cultivars that showed BN symptoms for this study, along with the most prevalent weeds in the corresponding vineyards. The complex interactions that ‘*Ca.* P. solani’ has with grapevine varieties, weeds, and the vectors generate a wide genetic diversity in the population, which can be evaluated by an MLST analysis [45]. An MLST analysis was applied to several ‘*Ca.* P. solani’ studies; it was found to be useful to study and monitor the epidemiology of ‘*Ca.* P. solani’ [33,36,46,47,48,49].

Our investigations were undertaken based on *vmp*1 and *stamp* multilocus sequence typing. These genes are linked to the biological aspects of ‘*Ca*. P. solani’ because the VMP and AMP proteins are both involved in the interactions between the phytoplasma and their vectors [50,51,52]. These two genes, therefore, have important roles in understanding the ecology of this pathogen; mutations in their sequences are strongly dependent on geographical distribution and the host range, which might be driven by their interactions with local vector(s) [30]. The diversity of ‘*Ca*. P. solani’ strains in Iranian vineyards was determined through the study of the variability of three genes linked to the epidemiology: *tuf*, *vmp*1, and *stamp*. Phylogenetic analyses of *vpm*1 and *stamp* highlighted that the genotypes within the clusters were related to the locations, providing useful information on the molecular epidemiology and the tracking of the ‘*Ca.* P. solani’ strain distribution.

According to the *vmp*1 typing, we distinguished six different profiles already reported in previous studies plus two new types—namely, V24 and V27—which have become prevalent in Iranian vineyards. A *stamp* gene phylogenetic analysis allowed the placement of most of the ‘*Ca*. P. solani’ strains in cluster II, which included samples from Central Europe and the Balkans (Germany, Czech Republic, Hungary, Croatia, and Bulgaria) [16,35], and cluster III, linking with Azerbaijan, Georgia, Lebanon, Serbia, Bosnia, and Herzegovina, and Austria strains. This confirmed that the ecology of ‘*Ca*. P. solani’ strains in Iran was associated with a *C. arvensis* reservoir. This was the case for the DW3 isolate, which belonged to cluster III. The grouping of the DW5 (*C. arvensis*), DW12 (*E. bonariensis*), and C6 (grapevine) strains in a different position related to cluster IV and FZ10, FZ11, and DG12 separately from the defined clusters considering their *vmp*1 type and *tuf* type might indicate the presence of interconnections between the cycles and/or that they might be linked to genetic recombination between the *stamp* clusters in Iranian vineyards.

Useful data were obtained from the genotyping of ‘*Ca*. P. solani’ in the viticultural regions of Iran, supporting the monitoring of the spread of this pathogen. The most common genotype on the grapevines was *tuf*-b/V24/*stamp* III, which is associated with *C. arvensis*. The important role of *C. arvensis* in the transfer of the *tuf*-b/V24/*stamp* III genotype to grapevines in vineyards was also identified in our investigation. The high genetic diversity among ‘*Ca*. P. solani’ strains indicated that the spread of BN is not through vegetative propagation, highlighting the important role of vectors in the spread of BN as well as the wild hosts as a source of infection. Considering the placement of DW5 and DW12 as wild hosts different from grapevine-associated ‘*Ca*. P. solani’ might indicate that further investigations are needed into different reservoir plants in Iranian vineyards. Tracing ‘*Ca*. P. solani’ in insect vectors and distinguishing possible further routes of its introduction into the grapevine ecosystem is required.

## 5. Conclusions

‘*Ca*. P. solani’ typing is crucial in grapevine and reservoir plants to clarify the epidemiological cycle(s) of BN, reflecting the important role of insect vectors in disease dispersal. Our work provides information on the molecular variants of ‘*Ca*. P. solani’ in Iranian vineyards; six known *vmp*1 variants were found as well as two novel variants, V24 and V27. This information is useful for future investigations to more accurately understand the epidemiological cycle(s) of BN in Iranian vineyards, contributing to the management of the disease.

## 6. Patents

No patents are related to this work.

## Figures and Tables

**Table 1:** ‘*Candidatus* Phytoplasma solani’ nucleotide sequences used in the phylogenetic analysis of the *vmp*1 and *stamp* genes from the investigated vineyards.

Isolate	Host	Origin	Accession Number	
			*vmp*1	*stamp*
a8	*Vitis vinifera*	Azarbaijan Gharbi	OM920494	
AG5	*Vitis vinifera*	Azarbaijan Gharbi	OM920495	
AG15	*Vitis vinifera*	Azarbaijan Gharbi	OM920496	OM791207
DG12	*Vitis vinifera*	Qazvin	MK547295	OM791210
DG23	*Vitis vinifera*	Zanjan	OM920497	OM791211
C4	*Vitis vinifera*	Zanjan	MK547294	OM791208
C6	*Vitis vinifera*	Qazvin	OM920498	OM791209
L3	*Vitis vinifera*	Lorestan	MK547300	OM791218
KG11	*Vitis vinifera*	Khorasan Razavi	MK547299	OM791217
FZ10	*Vitis vinifera*	Fars	MK547297	OM791215
FZ11	*Vitis vinifera*	Fars	MK547298	OM791216
DW3	*Convolvulus arvensis*	Qazvin	MK547296	OM791212
DW5	*Convolvulus arvensis*	Qazvin	OM920499	OM791213
DW12	*Erigeron bonariensis*	Zanjan	OM920500	OM791214

## Data Availability

The data presented in this study are openly available in the Sequence Read Archive of the National Center for Biotechnology Information (NCBI).

## Supplementary Materials

The following supporting information can be downloaded at: https://www.mdpi.com/article/10.3390/biology11060835/s1, Figure S1: Actual (right) and virtual (left) restriction fragment length polymorphism patterns of the representative vmp1 gene sequences amplified with the primer pair TYPH10F⁄R from the representative ‘*Candidatus* Phytoplasma solani’ strains. M 100 bp: Ladder, 100 bp (New England Biolabs); Table S1: Primer information for 16Sr DNA, *tuf*, *vmp*1, and *stamp* genes; Table S2: Detailed information about grapevine and weeds infected by ‘*Ca*. P. solani’ in Iranian vineyards.

## References

[1] Maixner M. (2011). Recent advances in Bois noir research. Petria.

[2] Quaglino F., Zhao Y., Casati P., Bulgari D., Bianco P.A., Wei W., Davis R.E. (2013). ‘*Candidatus* Phytoplasma solani’, a novel taxon associated with stolbur- and Bois noir-related diseases of plants. Int. J. Syst. Evol. Microbiol..

[3] Romanazzi G., Murolo S., Feliziani E. (2013). A new approach to manage phytoplasma diseases: Field treatments with resistance inducers to contain grapevine Bois noir. Phytopathology.

[4] Endeshaw S.T., Murolo S., Romanazzi G., Neri D. (2012). Effects of Bois noir on carbon assimilation, transpiration, stomatal conductance of leaves and yield of grapevine (*Vitis vinifera*) cv. Chardonnay. Physiol. Plant..

[5] Choueiri E., Jreijiri F., El Zammar S., Verdin E., Salar P., Danet J.L., Bove J., Garnier M. (2002). First report of grapevine “Bois Noir” disease and a new phytoplasma infecting solanaceous plants in Lebanon. Plant Dis..

[6] Duduk B., Tian J., Contaldo N., Fan X., Paltrinieri S., Chen Q., Zhao Q., Bertaccini A. (2010). Occurrence of phytoplasmas related to stolbur and to ‘*Candidatus* Phytoplasma japonicum’ in woody host plants in China. J. Phytopathol..

[7] Contaldo N., Soufi Z., Bertaccini A. (2011). Preliminary identification of phytoplasmas associated with grapevine yellows in Syria. Bull. Insectol..

[8] Quaglino F., Maghradze D., Chkhaidze N., Casati P., Failla O., Bianco P.A. (2014). First report of ‘*Candidatus* Phytoplasma solani’ and ‘*Ca*. P. convolvuli’ associated with grapevine Bois noir and bindweed yellows, respectively, in Georgia. Plant Dis..

[9] Salem N.M., Quaglino F., Abdeen A., Casati P., Bulgari D., Alma A., Bianco P.A. (2013). First report of ‘*Candidatus* Phytoplasma solani’ strains associated with grapevine Bois noir in Jordan. Plant Dis..

[10] Balakishiyeva G., Mammadov A., Foissac X., Huseynova I., Aliyev J. (2016). First report of grapevine ‘Bois noir’ in Azerbaijan. Plant Dis..

[11] Ertunc F., Orel D.C., Bayram S., Paltrinieri S., Bertaccini A., Topkaya S., Soylemezoglu G. (2015). Occurrence and identification of grapevine phytoplasmas in main viticultural regions of Turkey. Phytoparasitica.

[12] Mirchenari S.M., Massah A., Zirak L. (2015). ‘Bois noir’: New phytoplasma disease of grapevine in Iran. J. Plant Prot. Res..

[13] Sharon R., Harari A.R., Zahavi T., Raz R., Dafny-Yelin M., Tomer M., Sofer-Arad C., Weintraub P.G., Naor V. (2015). A yellows disease system with differing principal host plants for the obligatory pathogen and its vector. Plant Pathol..

[14] Porotikova E., Vinogradova S., Yurchenko E. (2019). Molecular identification of phytoplasmas in Russian vineyards. Phytopathog. Mollicutes.

[15] Maixner M. (1994). Transmission of German grapevine yellows (Vergilbungskrankheit) by the planthopper *Hyalesthes obsoletus* (Auchenorrhyncha: Cixiidae). Vitis.

[16] Cvrković T., Jović J., Mitrović M., Krstić Q., Toševski I. (2014). Experimental and molecular evidence of *Reptalus panzeri* as a natural vector of Bois noir. Plant Pathol..

[17] Gatineau F., Larrue J., Clair D. (2001). A new natural planthopper vector of stolbur phytoplasma in the genus *Pentastiridius* (Hemiptera: Cixiidae). Eur. J. Plant Pathol..

[18] Riedle-Bauer M., Sára A., Regner F. (2008). Transmission of a stolbur phytoplasma by the Agalliinae leafhopper *Anaceratagallia ribauti* (Hemiptera, Auchenorrhyncha, Cicadellidae). J. Phytopathol..

[19] Minuz R.L., Isidoro N., Casavecchia S., Burgio G., Riolo P. (2013). Sex-dispersal differences of four phloem-feeding vectors and their relationship to wild-plant abundance in vineyard agroecosystems. J. Econ. Entomol..

[20] Conigliaro G., Jamshidi E., Lo Verde G., Bella P., Mondello V., Giambra S., D’Urso V., Tsolakis H., Murolo S., Burruano S. (2020). Epidemiological investigations and molecular characterization of ‘*Candidatus* Phytoplasma solani’ in grapevines, weeds, vectors and putative vectors in western Sicily (Southern Italy). Pathogens.

[21] Jamshidi E., Murolo S., Corsi L., Landi L., Ruschioni S., Romanazzi G., Isidoro N., Riolo P. Identification and characterization of ‘*Candidatus* Phytoplasma solani’ associated with selected Auchenorrhyncha species. Proceedings of the VIII National Meeting on Phytoplasmas and Phytoplasmas Diseases.

[22] Quaglino F., Sanna F., Moussa A., Faccincani M., Passera A., Casati P., Bianco P.A., Mori N. (2019). Identification and ecology of alternative insect vectors of ‘*Candidatus* Phytoplasma solani’ to the grapevine. Sci. Rep..

[23] Lee I.-M., Martini M., Marcone C., Zhu S.F. (2004). Classification of phytoplasma strains in the elm yellows group (16SrV) and proposal of ‘*Candidatus* Phytoplasma ulmi’ for the phytoplasma associated with elm yellows. Int. J. Syst. Evol. Microbiol..

[24] Arnaud G., Malembic-Maher S., Salar P. (2007). Multilocus sequence typing confirms the close genetic interrelatedness of three distinct “Flavescence dorée” phytoplasma strain clusters and group 16SrV phytoplasmas infecting grapevine and alder in Europe. Appl. Environ. Microbiol..

[25] Danet J.L., Balakishiyeva G., Cimerman A., Sauvion N., Marie-Jeanne V., Labonne G., Lavina A., Batlle A., Krizanac I., Skoric D. (2011). Multilocus sequence analysis reveals the genetic diversity of European fruit tree phytoplasmas and supports the existence of inter-species recombination. Microbiology.

[26] Johannesen J., Foissac X., Kehrli P., Maixner M. (2012). Impact of vector dispersal and host-plant fidelity on the dissemination of an emerging plant pathogen. PLoS ONE.

[27] Langer M., Maixner M. (2004). Molecular characterization of grapevine yellows associated phytoplasmas of the stolbur-group based on RFLP-analysis of non-ribosomal DNA. Vitis.

[28] Murolo S., Mancini V., Romanazzi G. (2013). Spatial and temporal stolbur population structure in a cv. Chardonnay vineyard according *tovmp1* gene characterization. Plant Pathol..

[29] Aryan A., Brader G., Mörtel J., Pastar M., Riedle-Bauer M. (2014). An abundant ‘*Candidatus* Phytoplasma solani’ tuf b strain is associated with grapevine, stinging nettle and *Hyalesthes obsoletus*. Eur. J. Plant Pathol..

[30] Fabre A., Danet J.L., Foissac X. (2011). The stolbur phytoplasma antigenic membrane protein gene stamp is submitted to diversifying positive selection. Gene.

[31] Cimerman A., Pacifico D., Salar P., Marzachi’ C., Foissac X. (2009). Striking diversity of *vmp*1, a variable gene encoding a putative membrane protein of the stolbur phytoplasma. Appl. Environ. Microbiol..

[32] Pacifico D., Alma A., Bagnoli B., Foissac X., Pasquini G., Tessitori M., Marzachì C. (2009). Characterization of Bois noir isolates by restriction fragment length polymorphism of a stolbur-specific putative membrane protein gene. Phytopathology.

[33] Balakishiyeva G., Bayramova J., Mammadov A., Salar P., Danet J.-L., Ember I., Verdin E., Foissac X., Huseynova I. (2018). Important genetic diversity of ‘*Candidatus* Phytoplasma solani’ related strains associated with Bois noir grapevine yellows and planthoppers in Azerbaijan. Eur. J. Plant Pathol..

[34] Jamshidi E., Murolo S., Salehi M., Romanazzi G. (2020). Sequence analysis of new *Tuf* molecular types of ‘*Candidatus* Phytoplasma solani’ in Iranian vineyards. Pathogens.

[35] Foissac X., Carle P., Fabre A., Salar P., Danet J.L., STOLBUR-EUROMED Consortium ‘*Candidatus* Phytoplasma solani’ genome project and genetic diversity in the Euro–Mediterranean basin. Proceedings of the 3rd European Bois Noir Workshop.

[36] Murolo S., Romanazzi G. (2015). In-vineyard population structure of ‘*Candidatus* Phytoplasma solani’ using multilocus sequence typing analysis. Infect. Genet. Evol..

[37] Angelini E., Constable F., Duduk B., Fiore N., Quaglino F., Bertaccini A. (2018). Grapevine phytoplasmas. Phytoplasmas. Plant Pathogenic Bacteria-I.

[38] FAOSTAT, FAO Statistics Division (2019). Food and Agriculture Organization of the United Nations. http://www.fao.org/faostat/en/#data/QC.

[39] Jamshidi E., Murolo S., Ravari S.B., Salehi M., Romanazzi G. (2019). Molecular typing of ‘*Candidatus* Phytoplasma solani’ in Iranian vineyards. Plant Dis..

[40] Maixner M., Ahrens U., Seemüller E. (1995). Detection of the German grapevine yellows (Vergilbungskrankheit) MLO in grapevine, alternative hosts and a vector by a specific PCR procedure. Eur. J. Plant Pathol..

[41] Fialová R., Válová P., Balakishiyeva G., Danet J.L., Šafárová D., Foissac X., Navrátil M. (2009). Genetic variability of stolbur phytoplasma in annual crop and wild plant species in south Moravia. J. Plant Pathol..

[42] Fabre A., Balakishiyeva G., Ember I., Omar A., Acs Z., Kölber M., Kauzner L., Della Bartola M., Danet J.-L., Foissac X. (2011). Stamp encoding the antigenic membrane protein of stolbur phytoplasma is useful for molecular epidemiology. Bull. Insectol..

[43] Kumar S., Stecher G., Tamura K. (2016). MEGA7: Molecular Evolutionary Genetics Analysis version 7.0 for bigger datasets. Mol. Biol. Evol..

[44] Nei M., Kumar S. (2000). Molecular Evolution and Phylogenetics.

[45] Urwin R., Maiden M.C. (2003). Multi-locus sequence typing: A tool for global epidemiology. Trends Microbiol..

[46] Quaglino F., Maghradze D., Casati P., Chkhaidze N., Lobjanidze M., Ravasio A., Passera A., Venturini G., Failla O., Bianco P.A. (2016). Identification and characterization of new ‘*Candidatus* Phytoplasma solani’ strains associated with Bois noir disease in *Vitis vinifera* L. cultivars showing a range of symptom severity in Georgia, the Caucasus Region. Plant Dis..

[47] Kosovac A., Radonjić S., Hrnčić S., Krstić O., Toševski I., Jović J. (2016). Molecular tracing of the transmission routes of Bois noir in Mediterranean vineyards of Montenegro and experimental evidence for the epidemiological role of *Vitex agnus-castus* (Lamiaceae) and associated *Hyalesthes obsoletus* (Cixiidae). Plant Pathol..

[48] Delić D., Balech B., Radulović M., Lolić B., Karacić A., Vukosavljevic V., Ðurić G., Cvetković T.J. (2015). *Vmp*1 and *stamp* genes variability of ‘*Candidatus* phytoplasma solani’ in Bosnian and Herzegovinian grapevine. Eur. J. Plant Pathol..

[49] Pierro R., Passera A., Panattoni A., Casati P., Luvisi A., Rizzo D., Bianco P.A., Quaglino F., Materazzi A. (2018). Molecular typing of ‘Bois noir’ phytoplasma strains in the Chianti Classico area (Tuscany, Central Italy) and their association with symptom severity in *Vitis vinifera* L. cv. Sangiovese. Phytopathology.

[50] Suzuki S., Oshima K., Kakizawa S., Arashida R., Jung H.Y., Yamaji Y., Nishigawa H., Ugaki M., Namba S. (2006). Interaction between the membrane protein of a pathogen and insect microfilament complex determines insect-vector specificity. Proc. Natl. Acad. Sci. USA.

[51] Arricau-Bouvery N., Duret S., Dubrana M.P., Batailler B., Desqué D., Béven L., Danet J.L., Monticone M., Bosco D., Malembic-Maher S. (2018). Variable membrane protein A of Flavescence dorée phytoplasma binds the midgut perimicrovillar membrane of *Euscelidius variegatus* and promotes adhesion to its epithelial cells. Appl. Environ. Microbiol..

[52] Arricau-Bouvery N., Duret S., Dubrana M.P., Desqué D., Eveillard S., Brocard L., Malembic-Maher S., Foissac X. (2021). Interactions between the Flavescence dorée phytoplasma and its insect vector indicate lectin-type adhesion mediated by the adhesin VmpA. Sci. Rep..

