# Supplementary material for: Multilocus Genotyping of ‘Candidatus Phytoplasma Solani’ Associated with Grapevine Bois Noir in Iran
Source: Biology (Basel). 2022 May 29;11(6):835. doi: 10.3390/biology11060835 (PMC9220303; doi:10.3390/biology11060835)
Supplement: Supplementary file 1 [file biology-11-00835-s001.zip › biology-1658817-supplementary.pdf]

Supplementary Figure S1. Actual (right) and virtual (left) restriction fragment length polymorphism patterns of the representative vmp1 gene sequences amplified with the primer pair TYPH10F/R from the representative '*Candidatus* Phytoplasma solani' strains. M 100 bp: Ladder, 100 bp (New England Biolabs).

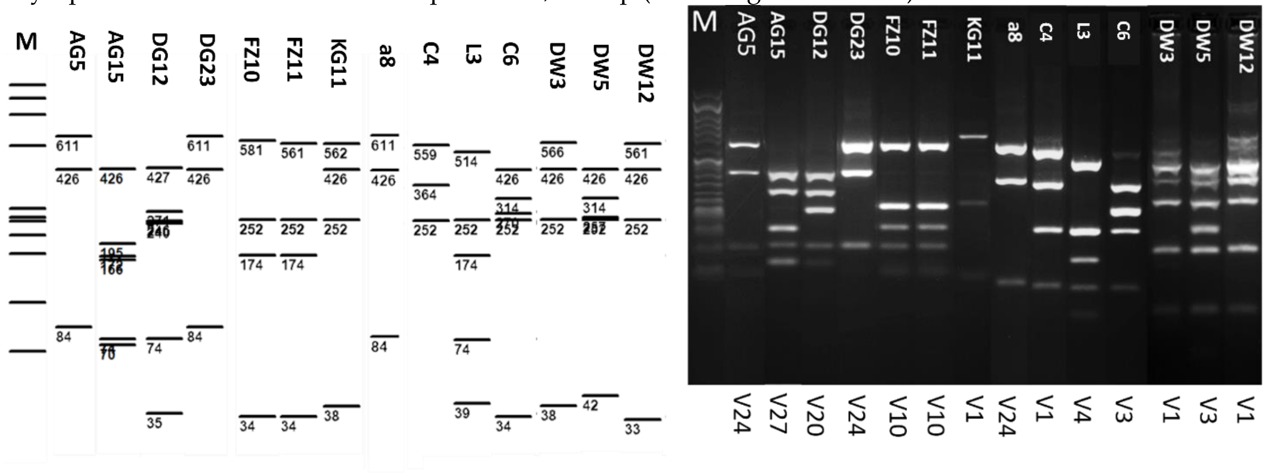

Supplementary Table S1. List of primers used for amplification of 16Sr DNA, *tuf*, *vmp*1 and *stamp* genes

Primer	Sequence	Reference
P1	AAGAGTTTGATCCTGGCTCAGGATT	Maixner et al., 1995
P7	GGATGGATCACCTCCTT	Maixner et al., 1995
fstol	GCCATCATTAAGTTGGGGA	Maixner et al., 1995
rstol	AGATGTGACCTATTTTGGTGG	Maixner et al., 1995
STOLTUF-F0	GCACGTTGATCACGGCAAAAC	Balakishiyeva et al., 2018
STOLTUFR0	CTGTTTTTCCACCTTCACGG	Balakishiyeva et al., 2018
fTufAY	GCTAAAAGTAGAGCTTATGA	Schneider et al., 1997
rTufAY	CGTTGTCACCTGGCATTACC	Schneider et al., 1997
StolH10F1	AGGTTGTAAAATCTTTTATGT	Cimerman et al., 2009
StolH10R1	GCGGATGGCTTTTCATTATTTGAC	Cimerman et al., 2009
StolH10F2	GGATTGATAGATGCTGCCCC	Cimerman et al., 2009
StolH10R2	CCATCAAAACTTTTTGGTTTAGGC	Cimerman et al., 2009
TYPH1OF	AACGTTCATCAACAATCAGTC	Fialova et al., 2009
TYPH1OR	CACTTCTTTCAGGCAACTTC	Fialova et al., 2009
StampF0	GTAGGTTTTGGATGTTTTAAG	Fabre et al., 2011
StampR0	AAATAAAAGAACAAGTATAGACGA	Fabre et al., 2011
StampF1	TTCTTTAAACACACCAAGAC	Fabre et al., 2011
StampR1	AAGCCAGAATTTAATCTAGC	Fabre et al., 2011

Supplementary Table S2. Detailed information about grapevine and weeds infected by '*Candidatus* Phytoplasma solani' in Iranian vineyards.

**Isolate**	**Host**	**variety**	**province**	**Molecular characterization**		
				***Tuf* type**	***vmp*1 profile**	***stamp***
AG1	*Vitis vinifera*	Qezel uzum	Azarbaijan Gharbi	b1	V24	
AG2	*Vitis vinifera*	Qezel uzum	Azarbaijan Gharbi	b1	V24	
AG4	*Vitis vinifera*	Qezel uzum	Azarbaijan Gharbi	b1	V24	
AG5	*Vitis vinifera*	Qezel uzum	Azarbaijan Gharbi	b1	V24	
AG6	*Vitis vinifera*	Qezel uzum	Azarbaijan Gharbi	b1	V24	
AG14	*Vitis vinifera*	Qezel uzum	Azarbaijan Gharbi	b1	V24	
AG15	*Vitis vinifera*	Qezel uzum	Azarbaijan Gharbi	b1	V27	Cluster III
a7	*Vitis vinifera*	Qermez Bidane Keshmeshi	Azarbaijan Gharbi	b1	V1	
a8	*Vitis vinifera*	Qermez Bidane Keshmeshi	Azarbaijan Gharbi	b5	V24	Cluster III
a11	*Vitis vinifera*	Qermez Bidane Keshmeshi	Azarbaijan Gharbi	b1	V24	
a13	*Vitis vinifera*	Qermez Bidane Keshmeshi	Azarbaijan Gharbi	b1	V24	
a20	*Vitis vinifera*	Qermez Bidane Keshmeshi	Azarbaijan Gharbi	b1	V4	
a22	*Vitis vinifera*	Qermez Bidane Keshmeshi	Azarbaijan Gharbi	b1	V4	
b1	*Vitis vinifera*	Khalili	Azarbaijan Sharghi	b1	V4	

BG2	*Vitis vinifera*	Khalili	Azarbaijan Sharghi	b1	V27	
b14	*Vitis vinifera*	Khalili	Azarbaijan Sharghi	b1	V4	
BG4	*Vitis vinifera*	Fakhri	Azarbaijan Sharghi	b1	V4	
BG9	*Vitis vinifera*	Fakhri	Azarbaijan Sharghi	b1	V1	
FG1	*Vitis vinifera*	Rish-baba	Fars	b1	V10	
FZ11	*Vitis vinifera*	Askari	Fars	b1	V10	
FZ10	*Vitis vinifera*	Askari	Fars	b1	V10	
KG10	*Vitis vinifera*	Peykami	Khorasan Razavi	b1	V1	
KG11	*Vitis vinifera*	Peykami	Khorasan Razavi	b1	V1	Cluster III
phG1	*Vitis vinifera*	Peykami	Khorasan Razavi	b1	V24	
phG2	*Vitis vinifera*	Peykami	Khorasan Razavi	b1	V24	
phG10	*Vitis vinifera*	Peykami	Khorasan Razavi	b1	V24	
L3	*Vitis vinifera*	Askari	Lorestan	b1	V4	Cluster III
LG17	*Vitis vinifera*	Sefide Bidane keshmeshi	Lorestan	b1	V27	
LG18	*Vitis vinifera*	Sefide Bidane keshmeshi	Lorestan	b1	V27	
LG19	*Vitis vinifera*	Sefide Bidane keshmeshi	Lorestan	b1	V27	
dG1	*Vitis vinifera*	Qermez Bidane Keshmeshi	Zanjan	b1	V24	

		Sefide Bidane keshmeshi				
dG5	*Vitis vinifera*	Sefide Bidane keshmeshi	Zanjan	b1	V3	
dG6	*Vitis vinifera*	Qermez Bidane Keshmeshi	Zanjan	b1	V24	
dG8	*Vitis vinifera*	Sefide Bidane keshmeshi	Zanjan	b1	V20	
dG9	*Vitis vinifera*	Qermez Bidane Keshmeshi	Zanjan	b1	V24	
C4	*Vitis vinifera*	Sefide Bidane keshmeshi	Zanjan	b1	V1	Cluster III
C6	*Vitis vinifera*	Qermez Bidane Keshmeshi	Zanjan	b1	V3	Cluster IV
dG10	*Vitis vinifera*	Qermez Bidane Keshmeshi	Zanjan	b1	V24	
dG11	*Vitis vinifera*	Sefide Bidane keshmeshi	Zanjan	b1	V24	
dG12	*Vitis vinifera*	Qermez Bidane Keshmeshi	Zanjan	b1	V20	Cluster III
DG19	*Vitis vinifera*	Qermez Bidane Keshmeshi	Qazvin	b1	V24	

DG20	*Vitis vinifera*	Qermez Bidane Keshmeshi	Qazvin	b1	V24	
DG21	*Vitis vinifera*	Qermez Bidane Keshmeshi	Qazvin	b1	V24	
DG22	*Vitis vinifera*	Qermez Bidane Keshmeshi	Qazvin	b1	V24	
DG23	*Vitis vinifera*	Qermez Bidane Keshmeshi	Qazvin	b6	V24	Cluster III
DW1	*C. arvensis*		Zanjan	b1	V24	
DW3	*C. arvensis*		Zanjan	b1	V1	Cluster III
DW5	*C. arvensis*		Zanjan	b1	V3	Cluster IV
DW7	*C. arvensis*		Qazvin	b1	V24	
DW11	*E. bonariensis*		Qazvin	b1	V3	
DW12	*E. bonariensis*		Qazvin	b1	V1	Cluster IV
AW2	*C. arvensis*		Azarbaijan Gharbi	b1	V1	
LW19	*C. arvensis*		Lorestan	b1	V15	
CoK1	*C. arvensis*		Khorasan Razavi	b1	V15	
